# Dynamic Incidence of Typhoid Fever over a 10-Year Period (2010–2019) in Kibera, an Urban Informal Settlement in Nairobi, Kenya

**DOI:** 10.4269/ajtmh.22-0736

**Published:** 2023-05-30

**Authors:** Eric Ng’eno, Margaret Lind, Allan Audi, Alice Ouma, Clifford Oduor, Patrick K. Munywoki, George O. Agogo, George Odongo, Samuel Kiplangat, Newton Wamola, Mike Powel Osita, Robert Mugoh, Caroline Ochieng, Victor Omballa, Ondari D. Mogeni, Matthew Mikoleit, Barry S. Fields, Joel M. Montgomery, Jillian Gauld, Robert F. Breiman, Bonventure Juma, Elizabeth Hunsperger, Marc-Alain Widdowson, Godfrey Bigogo, Eric D. Mintz, Jennifer R. Verani

**Affiliations:** ^1^ Centre for Global Health Research, Kenya Medical Research Institute, Nairobi, Kenya;; ^2^ Institute for Disease Modelling, Seattle, Washington;; ^3^ Department of Epidemiology of Microbial Diseases, Yale University School of Public Health, New Haven, Connecticut;; ^4^ Centre for Global Health Research, Kenya Medical Research Institute, Kisumu, Kenya;; ^5^ Division of Global Health Protection, Centers for Disease Control and Prevention, Nairobi, Kenya;; ^6^ Centers for Disease Control and Prevention, Atlanta, Georgia;; ^7^ Epidemiology, Public Health and Impact Unit, International Vaccine Institute, Seoul, South Korea;; ^8^ Department of Global Health, Rollins School of Public Health, Emory University, Atlanta, Georgia;; ^9^ Institute of Tropical Medicine, Antwerp, Belgium

## Abstract

Typhoid fever burden can vary over time. Long-term data can inform prevention strategies; however, such data are lacking in many African settings. We reexamined typhoid fever incidence and antimicrobial resistance (AMR) over a 10-year period in Kibera, a densely populated urban informal settlement where a high burden has been previously described. We used data from the Population Based Infectious Diseases Surveillance platform to estimate crude and adjusted incidence rates and prevalence of AMR in nearly 26,000 individuals of all ages. Demographic and healthcare-seeking information was collected through household visits. Blood cultures were processed for patients with acute fever or lower respiratory infection. Between 2010 and 2019, 16,437 participants were eligible for blood culture and 11,848 (72.1%) had a culture performed. Among 11,417 noncontaminated cultures (96.4%), 237 grew *Salmonella enterica* serovar Typhi (2.1%). Overall crude and adjusted incidences were 95 and 188 cases per 100,000 person-years of observation (pyo), respectively. Annual crude incidence varied from 144 to 233 between 2010 and 2012 and from 9 to 55 between 2013 and 2018 and reached 130 per 100,000 pyo in 2019. Children 5–9 years old had the highest overall incidence (crude, 208; adjusted, 359 per 100,000 pyo). Among isolates tested, 156 of 217 were multidrug resistant (resistant to chloramphenicol, ampicillin, and trimethoprim/sulfamethoxazole [71.9%]) and 6 of 223 were resistant to ciprofloxacin (2.7%). Typhoid fever incidence resurged in 2019 after a prolonged period of low rates, with the highest incidence among children. Typhoid fever control measures, including vaccines, could reduce morbidity in this setting.

## INTRODUCTION

Typhoid fever is a systemic, potentially life-threatening infection with *Salmonella enterica* serovar Typhi. An estimated 11 million typhoid fever infections and 117,000 deaths occur annually, primarily in resource-poor settings in Africa and Asia.^1^ Limited access to clean potable water and improved sanitation systems are important disease drivers in these settings.^2^^,^^3^ Residents living in densely populated urban informal settlements, which are often characterized by inadequate sanitation and drinking water, are at risk for both endemic and epidemic typhoid fever.^4^^–^^6^ Because an increasing proportion of the world’s population lives in informal settlements,^7^ understanding typhoid fever epidemiology in these settings is important for guiding control strategies.

The burden of typhoid fever is dynamic, varying across sites and within settings over time.^4^^,^^8^^–^^11^ Some urban locations have experienced substantial declines during the past decade,^12^^–^^14^ others have faced large, persistent outbreaks,^10^^,^^15^ and in some, the burden has largely remained unchanged.^16^ Rural settings have also faced high burdens of typhoid fever as well as outbreaks.^17^^,^^18^ The antimicrobial resistance (AMR) of *Salmonella* Typhi is also changing, with extensively drug resistant (XDR) strains exhibiting simultaneous resistance to fluoroquinolones, third-generation cephalosporins, ampicillin, chloramphenicol, and trimethoprim/sulfamethoxazole spreading globally.^19^

Understanding local typhoid fever epidemiology and AMR can inform the clinical management of patients with fever and guide decisions about preventive interventions such as typhoid vaccines, as well as sustainable investments in surveillance, water, sanitation, and hygiene (WASH). Although recent multicountry studies have generated valuable burden data in selected settings in Africa and Asia,^20^^–^^22^ long-term data on typhoid fever in urban sub-Saharan Africa are rare. Here, we describe the incidence, clinical characteristics, and AMR of typhoid fever infections over a 10-year period (2010–2019) in Kibera, an urban informal settlement in Nairobi, Kenya.^4^

## MATERIALS AND METHODS

### Study setting.

We used data from the Population-Based Infectious Disease Surveillance (PBIDS) platform in Kibera, Nairobi, operated by the Kenya Medical Research Institute, Center for Global Health Research (KEMRI-CGHR) in collaboration with the U.S. Centers for Disease Control and Prevention (CDC). Kibera is a large, densely populated urban informal settlement where residents have limited access to improved sanitation and clean drinking water.^23^^,^^24^

### Household and clinic surveillance.

The PBIDS methods have been previously described.^23^ Briefly, an enumerated population of ∼26,000 individuals of all ages were monitored through household visits and clinic-based surveillance. Individuals must have resided in the defined catchment area for a minimum of 4 months (or be born to a PBIDS participant) to be eligible to participate in the surveillance. During regular household visits, community interviewers collected self- and proxy-reported demographic information (births, deaths, in-migrations, and out-migrations) and data on recent illness and healthcare seeking. Household visits were conducted every 2 weeks between January and March 2010. The frequency was increased to weekly from April 2010 to April 2011 during the H1N1 influenza pandemic to optimize detection of H1N1 cases. Between May 2011 and April 2015, the frequency was reduced back to every 2 weeks and further to every 6 months from May 2015 to December 2019. However, household data collection tools and methods remained consistent; questions about illnesses and care seeking always refer to the 2 weeks before the interview.

Population-Based Infectious Disease Surveillance participants received free medical care for acute illness at the Tabitha Medical Clinic operated by Carolina for Kibera in collaboration with the KEMRI and CDC. All PBIDS households were located within an ∼1-km radius of the clinic.^24^ At the clinic, trained clinicians examined participants and identified those meeting criteria for acute lower respiratory illness (ALRI) or acute febrile illness (AFI). For children aged < 5 years, ALRI was defined as cough or difficulty breathing plus at least one of the following: chest indrawing, oxygen saturation < 90%, lethargy, convulsions, inability to drink fluids/breastfeed, vomiting everything, or stridor at rest. For individuals ≥ 5 years old, ALRI was defined as cough or difficulty breathing or chest pain plus an axillary temperature ≥ 38.0°C or oxygen saturation < 90%. For participants of all ages, AFI was defined as measured fever with onset of < 14 days. The definition of fever varied during the study period; starting at ≥ 38.0°C, the cutoff was decreased to ≥ 37.5°C in January 2012 and then raised to ≥ 38.0°C in January 2014. A single blood sample (1–3 mL in children < 5 years old and 8–10 mL for persons ≥ 5 years old) was collected for culture from consenting individuals meeting ALRI or AFI case definitions, before administration of antimicrobial agents when possible.^4^

### Laboratory procedures.

#### Blood culture.

Collected blood was inoculated into a BACTEC Ped Plus/F bottle (Temse, Belgium) (for children < 5 years old) or BACTEC Plus/F bottles (for individuals ≥ 5 years old). Inoculated blood culture bottles were incubated at 37°C for up to 5 days in a BACTEC 9050 system located at a KEMRI-CGHR laboratory in Kibera. Blood samples from alarm-positive bottles were plated on blood agar, chocolate agar, and MacConkey plates, and then incubated aerobically at 37°C for 24 hours. Gram stain and biochemical tests were used for identification,^25^ and serotyping was performed by the agglutination method using commercially produced group D and Vi antisera (Denka Seiken, Tokyo, Japan). In cases where isolates could not be serotyped by the agglutination method, the API 20E system (Biomérieux; appareils et procedures d’identification, Craponne, France) was used. Antimicrobial susceptibility testing (AST) was performed by Kirby-Bauer diffusion, using BD Difco antibiotic discs (Franklin Lakes, NJ).^26^ Antimicrobial susceptibility testing results were interpreted according to Clinical and Laboratory Standards Institute guidelines.^27^ We defined multidrug resistance (MDR) as resistance to chloramphenicol, ampicillin, and trimethoprim/sulfamethoxazole.

#### Whole-genome sequencing.

Sequencing was performed on 240 *Salmonella* isolates that could be revived from 2010 to February 2019. Of these, 198 (82.5%) were sequenced at Wellcome Sanger, 25 (10.4%) at Technical University of Denmark, and 17 (7.1%) at KEMRI-CGHR laboratories in Nairobi using previously described methods.^28^^–^^30^ Data quality was checked using the FastQC v0.11.15 tool.^31^ Assemblies were generated using Shovill (v1.0.4), excluding contigs with coverage below 30x. Assembled sequences were analyzed for multi-locus sequence type using BioNumerics v7.6.3 (Applied Maths, Austin, TX) and Enterobase scheme database (Achtman 7-gene MLST).^32^

### Data organization and statistical analysis.

We defined a typhoid fever case as *S*. Typhi bacteremia in a PBIDS participant. If *S*. Typhi was isolated more than once within a 14-day period from the blood of an individual, we considered it a single episode of typhoid fever and used data from the first isolation for analysis.

Crude annual typhoid fever incidence rates for 2010–2019 were calculated by dividing the annual number of typhoid fever cases by person-years of observation (pyo) among active PBIDS participants in that year.^4^ Each participant contributed person-time and clinic visits only during times of residency based on data from the periodic household visits. Enrolled participants were considered to have exited PBIDS if they were away from the study area for more than 4 consecutive months. For age-stratified incidence calculations, we used the following age groups: 0–1 years, 2–4 years, 5–9 years, 10–17 years, 18–24 years, 25–44 years, and more than 45 years.

We adjusted the incidence rates for probabilities of sample collection and healthcare seeking at the surveillance clinic. To adjust for sample collection, we divided the crude incidence by the proportion of all AFI and ALRI cases among active PBIDS participants seen in the clinic who had a blood culture performed. To adjust for healthcare seeking, we divided the incidence by the proportion of all medically attended ALRIs and AFIs reported in household surveys that had sought care at the Tabitha Medical Clinic surveillance facility (rather than other health facilities), based on recent illnesses reported during the interview.^4^ Adjustments were stratified by year and age group. When clinical information was missing, we assumed a distribution of the clinical syndromes similar to that in participants with clinical data in that year. Missing participants’ clinical presentation data occurred from failed linkage between clinic and laboratory data. We used smooth spline regression models to account for precision in risk estimation between age and time categories. Bootstrapping was used to account for uncertainty in adjustment factors (weights) and adjusted incidence rates. Analyses were performed in R software version 4.0.5.^33^ Smooth spline regression models were generated with the mgcv package mgcv version 1.8-31.^34^

#### Ethics statement.

The PBIDS protocol was approved by the KEMRI Scientific and Ethics Review Unit (SSC protocol number 1899 & 2761) and the CDC institutional review board (protocol number 4566 & 6775). Written informed consent was obtained from heads of households for their household members to participate in PBIDS. Individual household members could decline participation in the surveillance. At the clinic, additional individual written informed consent was obtained. For children aged < 7 years, parental consent was provided by the parent or guardian, whereas for children aged 7–17 years, parental consent and additional written assent were obtained from the child.

## RESULTS

**Figure 1.**
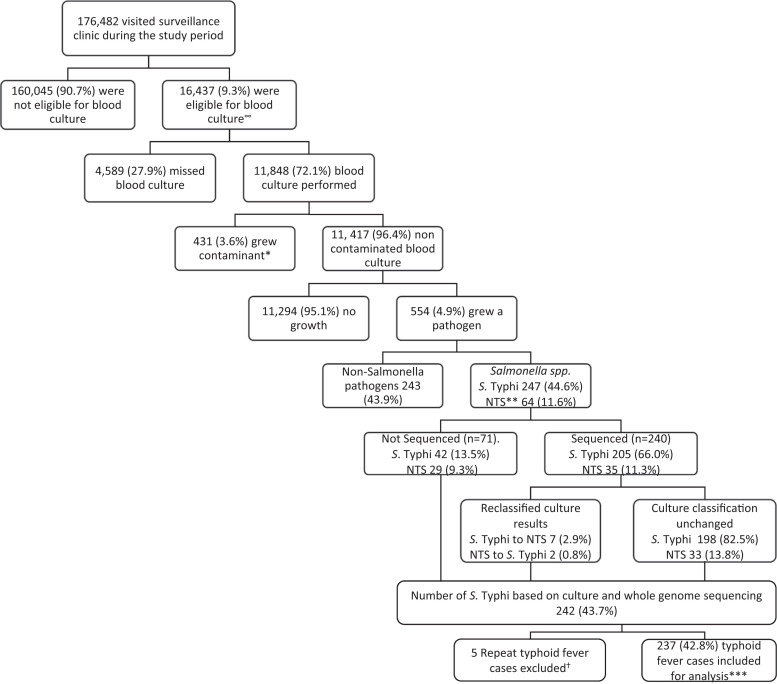
Flow diagram showing overall clinic visits, blood cultures performed, pathogens isolated, and sequenced *Salmonella enterica* serovar Typhi in Kibera, Nairobi, Kenya (2010–2019). ∞Number of those eligible for blood culture is adjusted for 1,022 blood cultures with missing clinical records. A distribution of clinical syndromes similar to that in participants with clinical data was assumed for these records in the different years. *Contaminants defined as *Staphylococcus* species coagulase negative, *Staphylococcus epidermidis*, *Staphylococcus xylosus*, gram-positive rods, *Micrococcus* species, *Serratia odorifera*, gram-positive* bacillus, and Erwinia* species. **NTS refers to non-typhoidal *Salmonella.* †Considered repeat if *S*. Typhi was isolated twice in < 14 days in the same individual. ***Denominator is blood cultures that grew a pathogen. *S*. Typhi isolation was 2.1% overall among non-contaminated blood cultures.

Between January 1, 2010 and December 31, 2019, 176,482 participants visited the clinic. Of these, 16,437 were eligible for blood culture (9.3%) and 11,848 had a blood culture performed (72.1%) (Figure 1). Among 11,417 noncontaminated blood cultures (96.4%), 554 grew a pathogen (4.9%), including 247 *S.* Typhi (44.6%) and 64 non-typhoidal *Salmonella* (NTS) (11.6%). Among 240 sequenced *Salmonella* spp., 7 *S.* Typhi (2.9%) were reclassified as NTS and 2 NTS (3.1%) were reclassified as *S.* Typhi, resulting in 242 *S.* Typhi (43.7%). All *S.* Typhi genomes belonged to the H58 (4.3.1) genotype. *S.* Typhi grew twice in five individuals within 14 days; these were considered single infections, leaving 237 (42.8%) unique typhoid fever cases.

The median age of typhoid fever cases was 8.4 years (interquartile range, 5.3–15.1 years); 50.6% were male (Table 1). Among 201 cases with any clinical data (84.8%), the most frequent signs and symptoms were measured axillary temperature ≥ 38.0°C (198 of 201 [98.5%]); reported fever (187 of 199 [94.0%]); headache 105 of 171, [61.4%]; cough 79 of 199 [39.7%]; chills 54 of 173 [31.2%]; abdominal pain 44 of 170 [25.9%]; and joint pains 39 of 171 [22.8%]. Among the 182 cases who received medication at the clinic, 121 were treated with an antimicrobial (66.5%), 15 with an antimalarial (8.2%), and 3 with both antimicrobial and antimalarial (1.6%), and 43 received an analgesic only (23.6%). Of 121 cases empirically prescribed antibiotics, amoxicillin, (47 [38.8%]) was most common, followed by amoxicillin/clavulanic acid 18 [14.9%], cephalexin 14 [11.6%], and ciprofloxacin 11 [9.1%]. Among 201 cases with data on clinical disposition, 1 was hospitalized (0.5%), and the remainder were managed as outpatients. Based on demographic data collected through household-based surveillance, no deaths were recorded among cases within 30 days of the date of blood culture.

**Table 1:** Demographics, clinical presentation, diagnosis, and treatment* of typhoid fever cases in Kibera, Nairobi, Kenya (2010–2019)

Characteristics	*n* (%)
Age (*N* = 237)	
Under 2 years	14 (5.9)
2–4 years	39 (16.5)
5–9 years	80 (33.8)
10–17 years	52 (21.9)
18–24 years	21 (8.9)
25–44 years	28 (11.8)
45 years and above	3 (1.3)
Male sex	120 (50.6)
Clinical presentation†	
Fever (measured temp °C ≥ 38.0)	198 (98.5)
Reported fever	187 (94.0)
Headache	105 (61.4)
Cough	79 (39.7)
Chills	54 (31.2)
Abdominal pain	44 (25.9)
Joint pain	39 (22.8)
Vomiting	31 (15.6)
Muscle pain	30 (17.5)
Reported diarrhea‡	30 (15.1)
Chest pain	10 (5.1)
Clinical diagnosis at surveillance clinic (*N* = 134)§	
Upper respiratory tract infection	45 (33.6)
Pneumonia	21 (15.7)
Malaria	13 (9.7)
Urinary tract Infection	12 (9.0)
Acute febrile illness	9 (6.7)
Gastroenteritis	8 (6.0)
Bacteremia	6 (4.5)
Amebiasis	5 (3.7)
Antimicrobials and antimalarials given at surveillance clinic (*N* = 182)	
Antimicrobials	121 (66.5)
Amoxycillin	47 (38.8)
Amoxiclav	18 (14.9)
Cephalexin	14 (11.6)
Ciprofloxacin	11 (9.1)
Erythromycin	8 (6.6)
Cefuroxime	4 (3.3)
Ceftriaxone	3 (2.5)
Ciprofloxacin and metronidazole	4 (3.3)
Erythromycin and metronidazole	2 (1.7)
Others	10 (8.3)
Antimalarials	15 (8.2)
Artemether/lumefantrine	12 (80.0)
Artemether/lumefantrine and antimicrobial	3 (1.6)
Analgesic	43 (23.6)

* Treatment was based on initial presentation, before culture results were available.

† Denominators are signs and symptoms that were examined, including measured temp °C ≥ 38.0 (201), reported fever (199), headache (171), cough (199), chills (173), abdominal pain (170), joint pain (171), vomiting (199), muscle pain (171), reported diarrhea (199), and chest pain (196).

‡ Diarrhea was defined as three or more loose stools in less than 24 hours.

§ Clinicians were provided a list of predetermined clinical diagnoses to choose from, with nonlisted diagnosis collected as free text. This may have introduced bias for listed diagnoses. Typhoid fever was not among the listed diagnoses.

**Figure 2.**
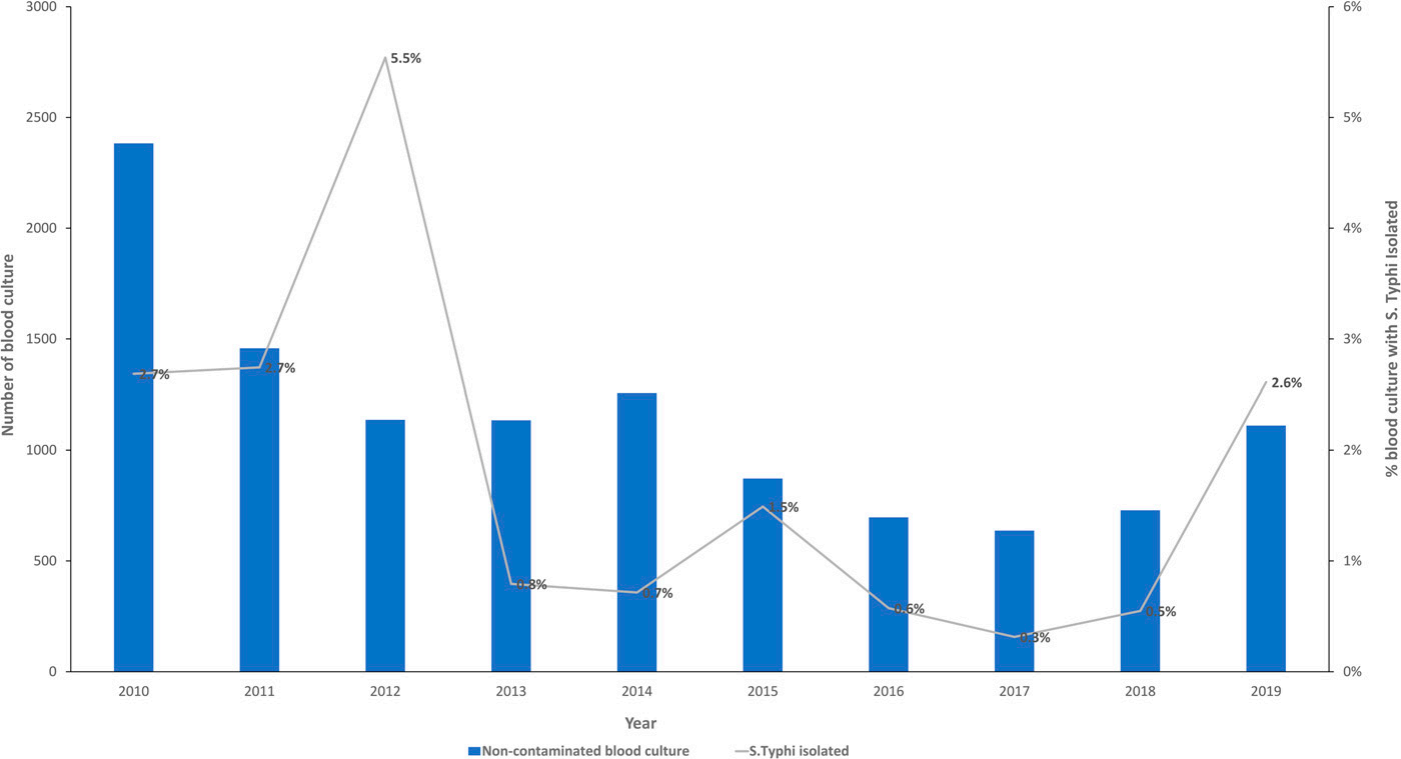
Proportion of *Salmonella enterica* serovar Typhi isolated from non-contaminated blood cultures by year at Tabitha Clinic, Kibera, Nairobi, Kenya, 2010–2019.

The proportion of noncontaminated blood cultures with *S*. Typhi isolated varied over time, with highest proportions in 2012 (63 of 1,145 [5.5%]), 2010 (64 of 2,370 [2.7%]), 2011 (40 of 1,481 [2.7%]), and 2019 (29 of 1,115 [2.6%]). The lowest *S*. Typhi isolation proportion was in 2017 (2 of 667 [0.3%]) (Figure 2).

### Crude and adjusted incidence rates.

**Figure 3.**
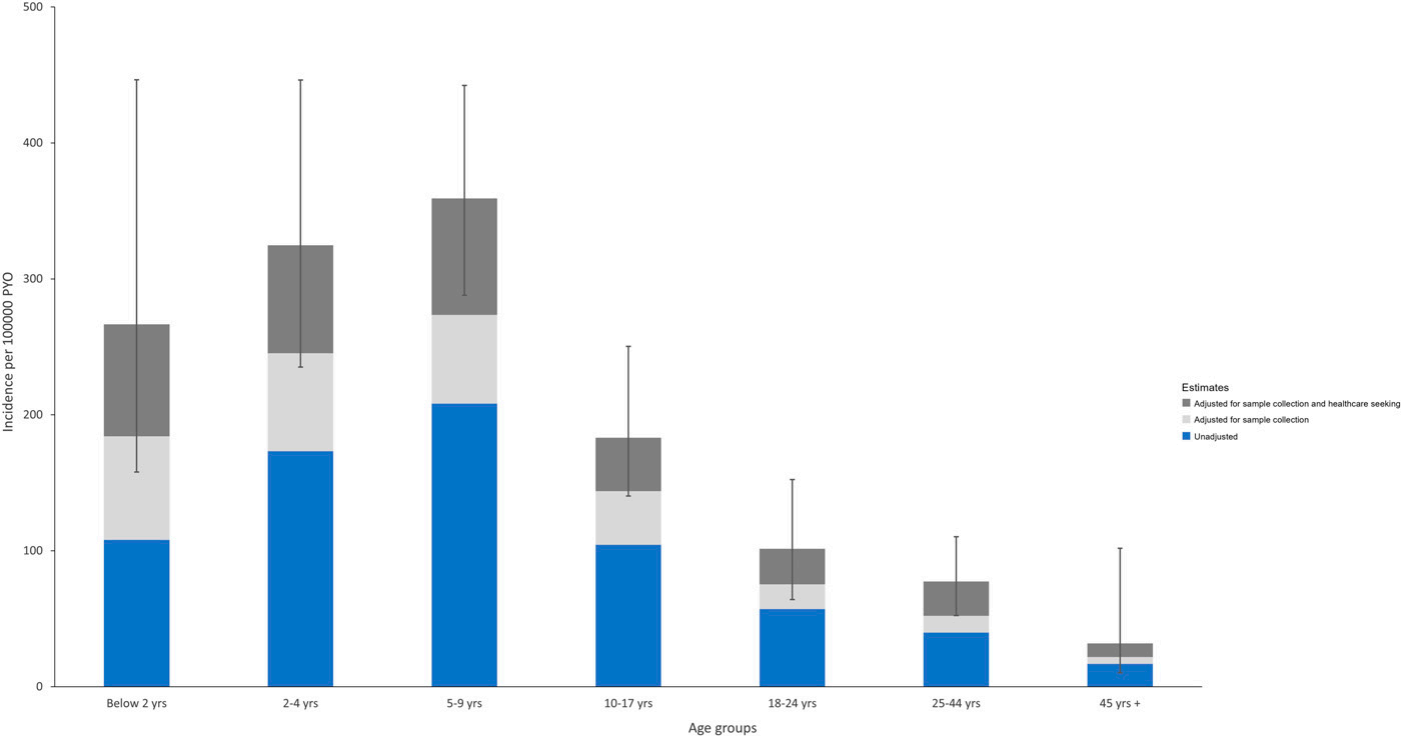
Distribution of crude and adjusted incidence rates of typhoid fever by age in Kibera, Nairobi, Kenya, 2010–2019. Bars reflect 95% CI for the incidence estimate adjusted for both sample collection and healthcare seeking. To adjust for sample collection, overall crude incidence for each age category was divided by the proportion of all acute febrile illness and acute lower respiratory illness cases among active PBIDS participants in the respective age category seen in the clinic who had a blood culture performed. To adjust for sample collection and healthcare seeking, the sample collection–adjusted estimate was divided by the proportion of all medically attended acute febrile illnesses and acute lower respiratory illnesses in the respective age category reported in household surveys that had sought care at the Tabitha Medical Clinic surveillance facility, based on illnesses reported during 14 days preceding a household interview. PBIDS = Population-Based Infectious Disease Surveillance; PYO = person-years of observation.

Overall, the crude and fully adjusted typhoid fever incidence rates during the study period were 95 per 100,000 pyo and 188 per 100,000 pyo, respectively (Table 2). The age group with the highest incidence was children aged 5–9 years (crude 208 per 100,000 pyo; adjusted 359 per 100,000 pyo), followed by 2–4 years (crude 173 per 100,000 pyo; adjusted 325 per 100,000 pyo) (Figure 3). Adults ≥ 45 years old had the lowest incidence (crude 17 per 100,000 pyo; adjusted 32 per 100,000 pyo).

**Table 2:** Bootstrapped overall crude and adjusted incidence rates of typhoid fever in Kibera, Nairobi, Kenya, 2010–2019

Syndromes	*S*. Typhi isolated	PYO	Crude incidence per 100,000 pyo (95% CI)	% Cultured	Blood culture adjusted (95% CI)	% Clinic visits	Blood culture and care seeking, adjusted (95% CI)
Overall	237	248,393.8	95.4 (84.0–108.4)	–	136.1 (118.0–152.8)	–	187.6 (162.6–211.0)
ALRI	60	–	–	72.4	–	74.8	–
AFI	139	–	–	70.2	–	66.4	–
Others*	38	–	–	–	–	–	–

AFI = acute febrile illness; ALRI = acute lower respiratory illness; PYO = person-years of observation.

* Includes blood culture on individuals not meeting criteria for ALRI or AFI. The blood cultures were performed for clinical reasons such as suspicion of sepsis.

**Figure 4.**
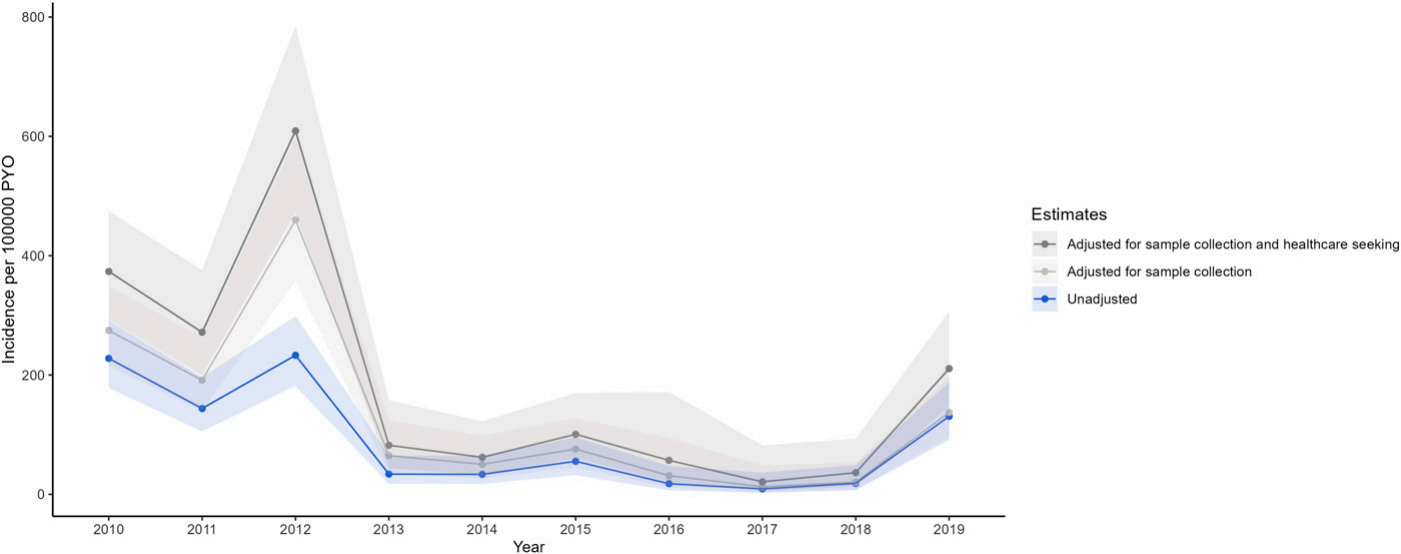
Crude and adjusted incidence trends of typhoid fever by year in Kibera, Nairobi, Kenya, 2010–2019. Shaded area reflects 95% confidence bands for the incidence estimates. Crude incidence was calculated by dividing the annual number of typhoid fever cases by person-years of observation among active PBIDS participants in that year. To adjust for sample collection, crude incidence was divided by the proportion of all acute febrile illness and acute lower respiratory illness cases among active PBIDS participants seen in the clinic who had a blood culture performed in that year. To adjust for sample collection and healthcare seeking, the sample collection–adjusted estimate was divided by the proportion of all medically attended acute febrile illnesses and acute lower respiratory illnesses reported in household surveys that had sought care at the Tabitha Medical Clinic surveillance facility in that year, based on illnesses reported during 14 days preceding a household interview. PBIDS = Population-Based Infectious Disease Surveillance; PYO = person-years of observation.

**Figure 5.**
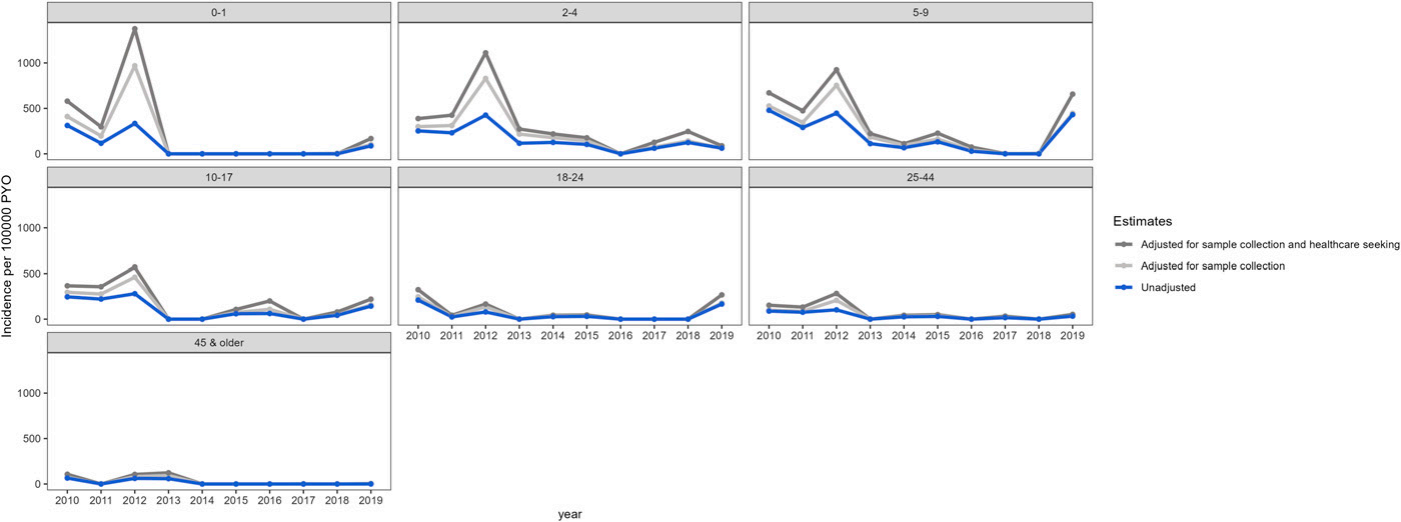
Crude and adjusted age group–stratified typhoid fever incidence by year in Kibera, Nairobi, Kenya, 2010–2019. Crude incidence was calculated by dividing the annual number of typhoid fever cases in each age category by person-years of observation among active PBIDS participants in the age category in that year. To adjust for sample collection, crude incidence was divided by the proportion of all acute febrile illness and acute lower respiratory illness cases among active PBIDS participants in the respective age category and year seen in the clinic who had a blood culture performed. To adjust for sample collection and healthcare seeking, the sample collection–adjusted estimate was divided by the proportion of all medically attended acute febrile illnesses and acute lower respiratory illnesses in the respective age category and year reported in household surveys that had sought care at the Tabitha Medical Clinic surveillance facility, based on illnesses reported during 14 days preceding a household interview. PBIDS = Population-Based Infectious Disease Surveillance; PYO = person-years of observation.

Annual crude incidence varied over time. Between 2010 and 2012, the crude annual incidence ranged from 144 to 233 per 100,000 pyo (Figure 4). Year-to-year decline in disease burden was observed from 2013 (34 per 100,000 pyo) to 2017 (9 per 100,000 pyo), with the exception of 2015, which had a slightly higher incidence (55 per 100,000 pyo) than 2014 (33 per 100,000 pyo). After the low point in 2017, the annual crude incidence increased to 18 per 100,000 pyo in 2018 and 130 per 100,000 in 2019. The adjusted overall annual incidence was highest in 2012 (608 per 100,000 pyo) and 2010 (375 per 100,000 pyo), whereas the lowest was observed in 2017 (20 per 100,000 pyo). The changes over time were most marked among children aged 2–4, 5–9, and 10–17 years and were relatively stable over time in adults 25 years and older (Figure 5).

### AMR.

**Figure 6.**
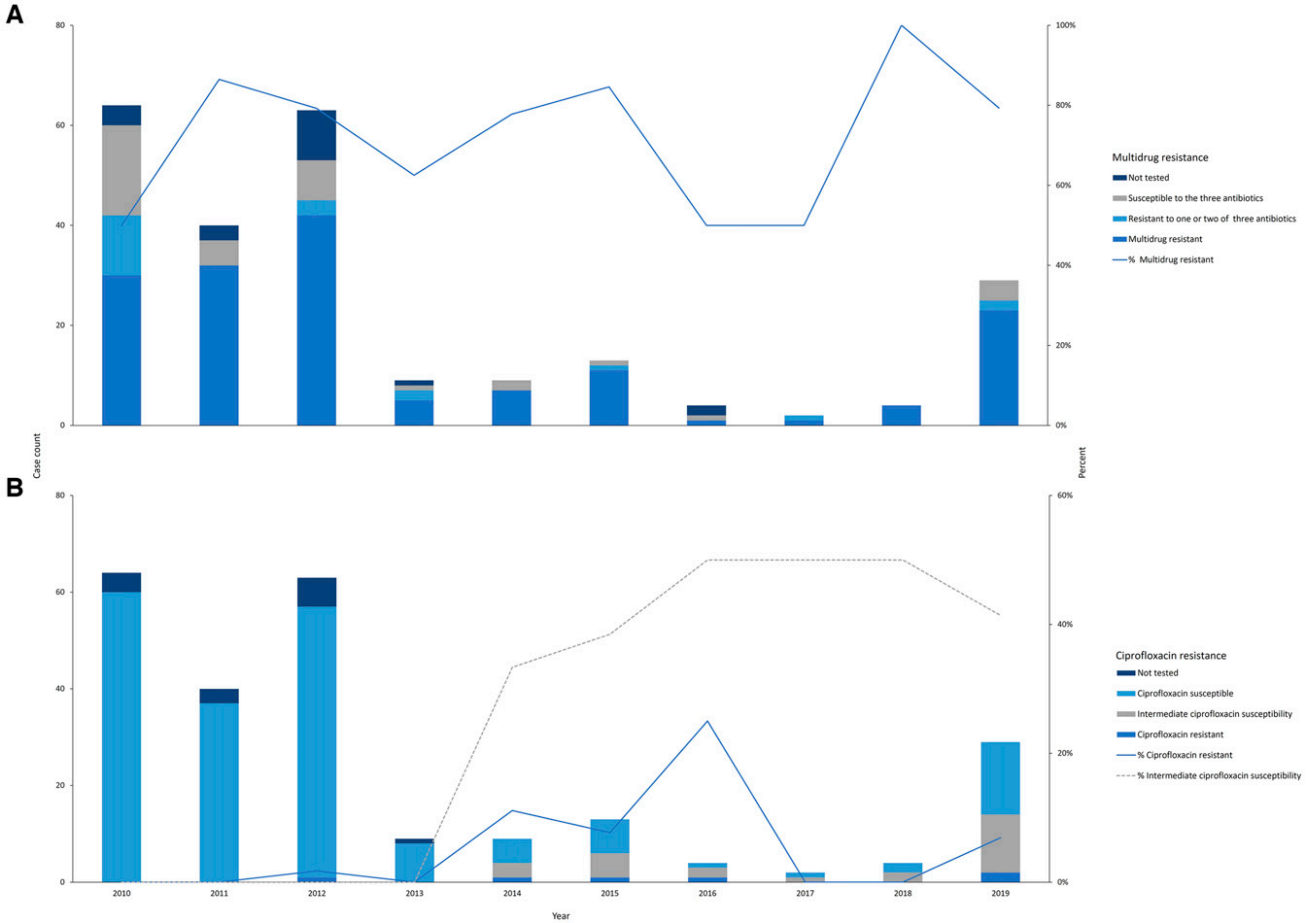
Multidrug resistance, ciprofloxacin resistance, and intermediate ciprofloxacin susceptibility over time among *Salmonella enterica* serovar Typhi blood culture isolates in Kibera, Nairobi, Kenya, 2010–2019. (**A** and **B**) Resistance proportion was calculated by dividing the number of *S. Typhi* isolates resistant to a specific antibiotic by the number of *S. Typhi* isolates tested for resistance to the antibiotic. Isolates were not tested for resistance where antibiotic disks were missing or when archived isolates could not be revived for later testing.

Among *S*. Typhi isolates that underwent AST, the prevalence of MDR was 71.9% (156 of 217). About half of *S*. Typhi isolates in each year were MDR (Figure 6A). Overall, the prevalence of ciprofloxacin resistance was 2.7% (6 of 223) and intermediate susceptibility was 11.2% (25 of 223); intermediate susceptibility increased from 2014 on (33.3–41.4% from 2014–2019) (Figure 6B). We did not observe isolates resistant to ceftriaxone.

## DISCUSSION

We observed a changing incidence of typhoid fever over time, as well as distinct age group–specific trends in incidence, prevalent MDR, sporadic ciprofloxacin resistance, and increasing intermediate susceptibility to ciprofloxacin. Data from the early years of this same surveillance platform (2007–2009) drew attention to a high burden (with a reported adjusted incidence of > 2,000 cases per 100,000 pyo) of typhoid fever in Kibera.^4^ With the benefit of additional years of data, we observed substantial variability in typhoid incidence over time, with peaks followed by a dramatic decline, followed by a resurgence of disease in the final year of this study. Similar to what was previously described in this setting,^4^ we observed the highest burden in children aged 2–9 years. Hospitalization was rare, which may reflect early detection of cases because of ready access to healthcare and testing. Although periods of declining disease burden are encouraging, our data illustrate that they may be relatively short-lived and not reflective of long-lasting environmental, behavioral, or immunological changes. Caution is advised when interpreting typhoid incidence over short periods; populations living in environmental conditions that increase typhoid fever risk may nonetheless have a low measured disease burden for a 5-year period or longer, particularly during years following a period of high incidence^35^. However, in the absence of substantive environmental or behavioral changes in WASH, food production, preparation, and consumption or a sustained introduction of effective typhoid vaccines in childhood immunization programs, the risk of a recrudescence of typhoid fever incidence likely remains high.

The findings of recent large, multicountry studies of typhoid fever burden in diverse settings in Africa and Asia help contextualize our results.^9^^,^^17^^,^^21^^,^^22^^,^^36^ The Typhoid Fever Surveillance in Africa Program (TSAP), which included the PBIDS Kibera site, reported incidences ranging from 10 per 100,000 in Bandim, an urban setting in Guinea-Bissau, to 383 per 100,000 in Polesgo, a semi-urban setting in Burkina Faso. Across all TSAP sites, the age group with the highest burden was 2–4 year-olds, but some variation across sites was observed, with some sites reporting the highest incidence among ages 5–14 years.^36^ The Strategic Typhoid Alliance across Africa and Asia study included an urban setting in Blantyre, Malawi, where an even higher typhoid incidence was estimated: an overall incidence of 444 per 100,000, with the highest incidence, 861 per 100,000, in children 5–9 years old. Urban sites in South Asia also reported higher incidences than what we observed in Kibera, with similar patterns across age groups.^22^ However, methodologic differences, such as adjustment for blood culture sensitivity, limit the ability to directly compare incidence across studies.^37^ Studies in other sub-Saharan African settings have reported typhoid fever case fatality proportions of 2–2.7%^10^^,^^22^^,^^38^; however, we observed only one hospitalized case and no deaths, consistent with what has been previously reported from this platform,^4^ likely reflecting the outpatient setting and ready access to medical care, which facilitates early detection of cases. Although the multisite studies of typhoid fever have covered periods of 17–24 months,^22^^,^^36^ our finding that typhoid incidence is dynamic, with long periods between changes in risk, highlights the importance of monitoring typhoid fever over many years, even after periods of relatively low incidence.

Various factors can influence the burden of typhoid fever. Typhoid burden can decrease with improvements in populations’ access to WASH.^39^^–^^41^ Recognition of clean drinking water as a basic human right in the 2010 Kenyan constitution obligated the government to provide legislative and policy measures to ensure universal access. Consequently, resource allocation was increased for Nairobi’s informal settlement regional office to coordinate efforts by the government and its partners to strengthen water and sanitation access in Nairobi urban informal settlements. In Kibera, the office has overseen expansion of treated water distribution networks, upgrading of piping systems, reduction of unauthorized connections, and monitoring of established infrastructure since 2014.^42^^–^^44^ An aerial water distribution system was also introduced in the study area in 2016.^45^ Although decline of typhoid fever burden in the area may be consistent with improvements in access to clean drinking water, the resurgence in 2019 could indicate a breakdown in the sanitation system or the role of other drivers of risk. Early detection and treatment of typhoid cases can disrupt disease transmission.^46^ Free medical care offered to PBIDS participants at the surveillance clinic (a facility with blood culture capability) could have increased early detection and treatment of cases, potentially reducing community transmission in subsequent years. In settings where infection is common, a decreasing pool of susceptible individuals due to naturally acquired (or vaccine-induced) immunity can lead to a decline in cases.^35^ Conversely, long periods of reduced incidence can increase the proportion of the population that is immunologically naive to the disease through birth and in-migration (dilution) and through waning immunity in previously infected individuals.^47^ If the pathogen is still circulating in chronic or convalescent carriers, as is often the case,^48^ or is reintroduced, this can lead to an increase in cases. The resurgence we observed in 2019 was primarily among younger age groups, and it is possible that these factors played a role. This observation highlights the importance of more definitive, long-term control strategies such as improvements in WASH and immunization programs.

Three types of typhoid vaccines are currently available: typhoid conjugate vaccines (TCV), unconjugated Vi polysaccharide vaccines, and live attenuated Ty21a vaccines.^49^ Although The World Health Organization (WHO) has recommended the latter two since 2008 for endemic and epidemic settings, the more recently available, WHO-prequalified TCVs offer greater promise to control typhoid fever in resource-poor settings such as Kibera. In contrast to other typhoid vaccines, TCVs are administered as a single-dose injection and are safe and immunogenic in children aged < 2 years (approved for use in those aged ≥ 6 months). Typbar TCV (Bharat Biotech, Hyderabad, India), which was prequalified in 2018, has shown 79–85% efficacy against typhoid fever in phase 3 trials,^50^^,^^51^ and effectiveness data for TYPHIBEV^®^ (Biological E, Hyderabad, India), a second TCV prequalified in 2020, is undergoing evaluation.^52^ Since 2018, Gavi, the Vaccine Alliance–eligible countries, have also been able to apply for Gavi funds to support Typbar TCV introduction.^53^ Typhoid conjugate vaccines can therefore be incorporated into routine infant immunization programs and/or administered through focused campaigns and provide a critical tool to address the high burden of disease we observed in Kibera, including among children aged 0–1 year.^54^^,^^55^ These data from a high-burden setting in Kenya provide a baseline against which TCV impact could be assessed. However, the dynamic burden of disease observed over a 10-year period with no targeted typhoid fever interventions highlights the complexities of future TCV impact evaluations.

As previously observed in this and other similar settings in Kenya, MDR was common throughout the study period.^4^^,^^56^ The high prevalence is likely due to dominance of genotype 4.3.1 (H58), which has been associated with the resistance.^48^^,^^57^^,^^58^ Multidrug-resistant *S*. Typhi remains prevalent in Africa despite indications of a decrease in Asia.^59^ Persistence of MDR strains has been attributed to common use of β-lactam antimicrobials in self-management of respiratory and nonspecific febrile illness and prophylactic use of co-trimoxazole antimicrobials.^60^^–^^63^ With widespread resistance to these traditional first-line antimicrobials, orally administered fluoroquinolones such as ciprofloxacin and ofloxacin are preferred antimicrobials for enteric fever management.^64^^–^^66^ Strains resistant to these antimicrobials have emerged and are spreading, resulting in some settings shifting to third-generation cephalosporins and macrolides for first-line typhoid fever management.^59^ Although case counts were relatively small in the latter part of the study period, we have observed an increase in isolates with decreased susceptibility to ciprofloxacin since 2013. Ceftriaxone resistance was not observed; nonetheless, XDR *S.* Typhi (a variant that is MDR and resistant to both fluoroquinolones and ceftriaxone) is a growing global public health threat, and continued monitoring of antimicrobial susceptibility in Kibera is warranted.^67^^,^^68^

These findings are reported with the following limitations. A relatively small population was studied; variations in case counts due to chance could have impacted estimates of annual incidence rates. Although we adjusted for lack of sample collection and for healthcare seeking at non-surveillance facilities in our incidence calculations, persons non-sampled or seeking care at facilities other than Tabitha Clinic could differ in their risk for typhoid fever from those sampled. Also, additional factors that might have led to underestimation of disease burden, such as insensitivity of blood culture, prior antimicrobial use, volume of blood collected, and blood culture contamination, were not accounted for in our incidence calculations. Changes in frequency of household surveys from 2015 could have potentially decreased the accuracy of population estimates that were used as the denominator for incidence rates. Small numbers of isolates, particularly from 2013 onward, limited the interpretation of AMR patterns over time. Data on clinical diagnosis and treatment at initial presentation before culture results were missing for a substantial proportion of typhoid fever cases.

Our findings show a variable but persistently high burden of typhoid fever in Kibera, with the most recent data indicating an increasing trend. Introduction of TCV into childhood vaccination programs and catchup campaigns for older children could be prioritized to reduce burden in the highest risk group. However, to fully address typhoid fever in Kibera, long-term plans to improve standards of living, including ensuring adequate and equitable access to safe drinking water and safe sanitation facilities, are needed. A better understanding of the factors influencing the dynamic incidence could help refine control measures.
